# Supplementary material for: Splice-Junction-Based Mapping of Alternative Isoforms in the Human Proteome
Source: Cell Rep. Author manuscript; Available in PMC 2020 Jan 15. (PMC6961840; doi:10.1016/j.celrep.2019.11.026)
Supplement: 2 [file NIHMS1546469-supplement-2.zip › DF1/PXD010154/Testis/Testis_40_MLX_ANSIGSTSASSVPNTGR.pdf]

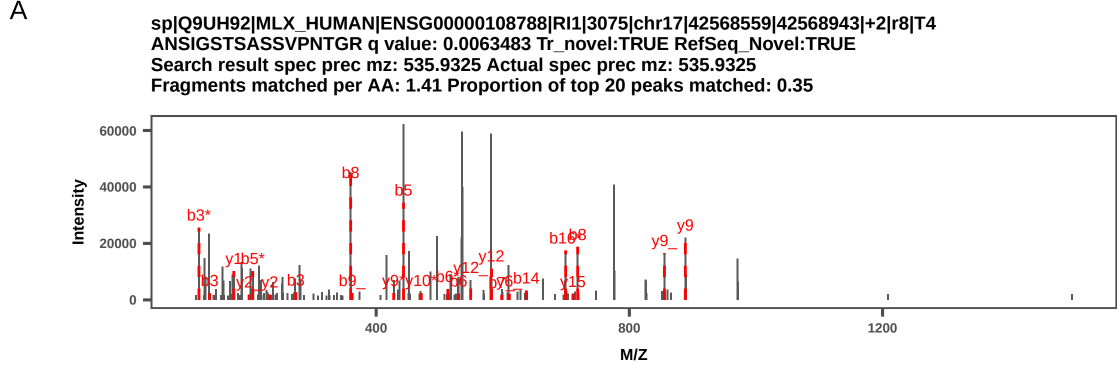
A
sp|Q9UH92|MLX_HUMAN|ENSG00000108788|RI1|3075|chr17|42568559|42568943|+2|r8|T4
ANSIGSTSASSVPNTGR q value: 0.0063483 Tr_novel:TRUE RefSeq_Novel:TRUE
Search result spec prec mz: 535.9325 Actual spec prec mz: 535.9325
Fragments matched per AA: 1.41 Proportion of top 20 peaks matched: 0.35
Intensity
60000
40000
20000
0
400
800
1200
M/Z

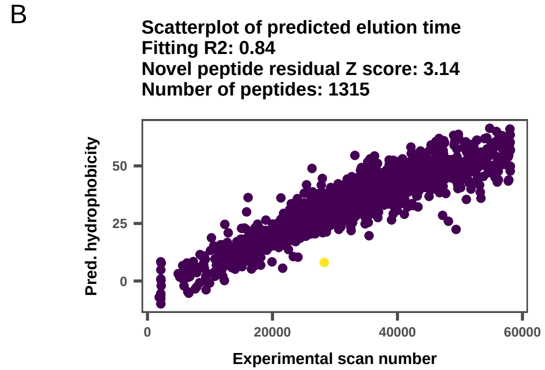
B
Scatterplot of predicted elution time
Fitting R2: 0.84
Novel peptide residual Z score: 3.14
Number of peptides: 1315
Pred. hydrophobicity
50
25
0
0
20000
40000
60000
Experimental scan number

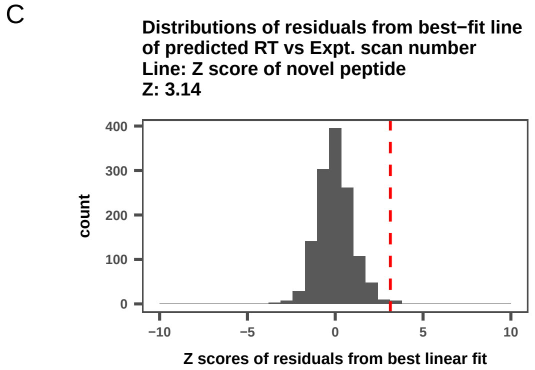
C
Distributions of residuals from best−fit line
of predicted RT vs Expt. scan number
Line: Z score of novel peptide
Z: 3.14
count
400
300
200
100
0
−10
−5
0
5
10
Z scores of residuals from best linear fit
